# Diverse effects of nitric oxide reductase NorV on *Aeromonas hydrophila* virulence-associated traits under aerobic and anaerobic conditions

**DOI:** 10.1186/s13567-019-0683-6

**Published:** 2019-09-23

**Authors:** Jin Liu, Yuhao Dong, Nannan Wang, Shuiyan Ma, Chengping Lu, Yongjie Liu

**Affiliations:** 1 Joint International Research Laboratory of Animal Health and Food Safety, College of Veterinary Medicine, Nanjing Agricultural University, Nanjing, 210095 China; 2 College of Animal Science and Technology, Jinling Institute of Technology, Nanjing, 211169 China

## Abstract

NorV has been known to be an anaerobic nitric oxide reductase associated with nitric oxide (NO) detoxification. Recently, we showed that the *norV* gene of *Aeromonas hydrophila* was highly upregulated after co-culturing with *Tetrahymena thermophila*. Here, we demonstrated that the transcription and expression levels of *norV* were upregulated in a dose-dependent manner after exposure to NO under aerobic and anaerobic conditions. To investigate the roles of *norV* in resisting predatory protists and virulence of *A. hydrophila*, we constructed the *norV* gene-deletion mutant (Δ*norV*). Compared to the wild type, the Δ*norV* mutant showed no significant difference in growth at various NO concentrations under aerobic conditions but significantly stronger NO-mediated growth inhibition under anaerobic conditions. The deletion of *norV* exhibited markedly decreased cytotoxicity, hemolytic and protease activities under aerobic and anaerobic conditions. Also, the hemolysin co-regulated protein (Hcp) in the Δ*norV* mutant showed increased secretion under aerobic conditions but decreased secretion under anaerobic conditions as compared to the wild-type. Moreover, the inactivation of *norV* led to reduced resistance to predation by *T. thermophila*, decreased survival within macrophages and highly attenuated virulence in zebrafish. Our data indicate a diverse role for *norV* in the expression of *A. hydrophila* virulence-associated traits that is not completely dependent on its function as a nitric oxide reductase. This study provides insights into an unexplored area of NorV, which will contribute to our understanding of bacterial pathogenesis and the development of new control strategies for *A. hydrophila* infection.

## Introduction

*Aeromonas hydrophila* is a Gram-negative bacterium, commonly found in a variety of natural aquatic environments worldwide including seawater, freshwater, sediments and even drinking water [1]. This bacterium is responsible for a variety of diseases in amphibians, fish and reptiles. As a major pathogen causing hemorrhagic septicemia in fish, *A. hydrophila* causes severe economic losses to aquaculture worldwide [2, 3]. The pathogenesis of *A. hydrophila* is complex and multifactorial, probably resulting from the expression of virulence factors such as adhesins, enterotoxins, hemolysin, aerolysin, and proteases, and as well as the secretion systems such as type III (T3SS) and type VI (T6SS) secretion systems [2–5].

Although many virulence factors have already been identified in *A. hydrophila*, several remain to be discovered. It is noteworthy that environmental factors, such as protistan predation, have an important impact on the virulence evolution of pathogens [6]. Protists may provide a protective reservoir for pathogens and act as a “training ground” for bacterial virulence [7, 8]. It has been demonstrated that pathogens including *Legionella pneumophila* [9], *Salmonella enteritidis* [10, 11], and *Mycobacterium avium* [12] are able to survive protistan predation, subsequently resulting in resistance to adverse situations such as antibiotics, oxidants and bioacids and increasing virulence. Grazing resistance to protists is an evolutionary precursor of bacterial pathogenicity and promotes bacteria to develop some defensive mechanisms for survival, such as new gene expression patterns or new proteins which may emerge as virulence determinants in animal and human infections [13, 14].

In our previous study, the *norV* gene of *A. hydrophila* was screened and identified to be about 15-fold upregulated under predation pressure of *Tetrahymena thermophila* [15]. The *norV* encodes a flavorubredoxin, a nitric oxide reductase, which is supposed to be inactivated by oxygen and provides physiological protection against nitric oxide (NO) only at low or zero oxygen concentrations [16]. The transcription of *norV* gene has been reported to be stimulated via a nitric oxide sensor NorR and sigma factor 54 (σ^54^)-dependent mechanism [17]. The binding of mononuclear iron site to NO activates the ATPase activity of NorR and enables NorR to interact with σ^54^-containing RNA polymerase to regulate the transcription of *norV* [18]. NorV, functioning as a major defense factor, contributes to bacterial resistance against oxidative and nitrosative killing [19–21]. Bacteria engulfed within the phagosomes of protists share similar defensive mechanisms responsible for survival within macrophages [22, 23]. During infection, bacterial cells are typically internalized into macrophages, enclosed in the phagolysosome and exposed to nitrogen radicals that are derived from inducible nitric oxide synthase (iNOS) [24]. NO is toxic to bacteria and usually deployed by macrophages as a potent antimicrobial for inhibition of pathogen proliferation, metabolic blockade, inactivation of virulence factors and dispersion of bacterial biofilm [25]. It has been reported that the *norV* mRNA expression was upregulated in *Salmonella enterica* sv. Typhimurium after macrophage internalization [26]. In addition, *norV* has been identified to limit NO level and contribute to the production of shiga toxin 2 (Stx2) of enterohaemorrhagic *Escherichia coli* (EHEC) within macrophages [27]. Whether and how *norV* gene is involved in stress response and pathogenicity of *A. hydrophila* are still unclear.

In this study, we evaluated the biological functions of the *norV* gene both in the aerobic and anaerobic environments. We also proposed a possible role of *norV* in *A. hydrophila* virulence and its resistance to predation by *T. thermophila*.

## Materials and methods

### Strains, cell lines and media

*Aeromonas hydrophila* strain NJ-35 (accession number CP006870) and *E. coli* SM10 were maintained in Luria–Bertani (LB) media at 28 °C and 37 °C, respectively. When required, media were supplemented with ampicillin (100 μg/mL), chloramphenicol (34 μg/mL) or gentamicin (100 μg/mL).

*Tetrahymena thermophila* SB210 (accession number GCA_000261185.1) was obtained from Dr. Miao Wei, Institute of Hydrobiology, China Academy of Sciences, and cultured in SPP medium (2% protease peptone, 0.1% yeast extract, 0.2% glucose, 0.003% EDTA-Fe) at 28 °C.

RAW264.7 cells were maintained in Dulbecco’s modified Eagle medium (DMEM; Gibco, New York, USA) supplemented with 10% (vol/vol) heat-inactivated fetal bovine serum (FBS; Gibco). All reagents used in this study were supplied by Sigma (St. Louis, MO, USA) unless otherwise indicated.

### Inactivation and complementation of *norV* gene in *A. hydrophila*

The *norV* gene was knocked out on the basis of the suicide plasmid pYAK1 via homologous recombination as previously described [28]. Firstly, the two flanking regions of *norV* were fused and cloned into pYAK1. The resulting plasmid was conjugated into *A. hydrophila* NJ-35 (resistance to ampicillin, Amp^r^) from *E. coli* SM10 and transconjugants were selected on LB agar plates with ampicillin and chloramphenicol. The positive colonies were cultured in LB medium without sodium chloride for 12 h and then counter-selected by growing on LB agar plates containing 20% sucrose. The gene-deletion mutant Δ*norV* was verified by PCR.

Genetic complementation was carried out by inserting the *norV* gene with a synonymous point mutation to the genome of Δ*norV* mutant. The *norV* gene and its two flanking regions were amplified from NJ-35 genomic DNA allowing a point mutation (G1236A) in *norV*. The fused PCR product was cloned into pYAK1 and conjugated into the Δ*norV* (Amp^r^). Chromosomal integration was achieved via allelic homologous recombination. The complemented strain CΔ*norV* was screened by antibiotics (ampicillin and chloramphenicol) and 20% sucrose in sequence and further verified by PCR and sequencing. The primers used are listed in Additional file 1.

### NO growth inhibition assay

NO growth inhibition assay was performed as previously described [27]. *A. hydrophila* wild-type, Δ*norV* and CΔ*norV* strains grown overnight were adjusted to OD_600_ of 1.0 with fresh LB media. The bacterial suspensions were diluted 1:100 with LB containing various concentrations of NO donor (sodium nitroprusside, SNP) and grown statically at 28 °C under aerobic or anaerobic conditions. Cultures without any treatment served as control. Then the OD_600_ values were measured every 1 h by a spectrophotometer (Bio-Rad, USA). Each sample was repeated in triplicate.

### Measurement of LDH release, hemolytic and protease activity

*Aeromonas hydrophila* wild-type, Δ*norV* and CΔ*norV* strains were cultured with or without NO donor to OD_600_ of 0.8 both under aerobic and anaerobic conditions. The culture supernatants were collected through centrifugation and filtration.

Cytotoxicity of RAW 264.7 macrophages induced by bacterial extracellular products (ECPs) was evaluated by measuring the release of lactate dehydrogenase (LDH) with a CytoTox 96 nonradioactive cytotoxicity assay (Promega, USA). The assay was performed according to the manufacturer’s instructions. Macrophages grown in 96-well plates were washed and added with 100 μL/well MEM containing 10 μL of the above mentioned culture supernatants. The plate was then incubated for 4 h at 37 °C with 5% CO_2_. LDH released by lysis of cells with 1% (vol/vol) Triton X-100 was defined as cell maximum release. LDH released by uninfected cells was designated as cell spontaneous release. The release of LDH was determined by measuring OD_492_ using a micro-plate reader (Tecan, Switzerland). Cytotoxicity was calculated as follows: % cytotoxicity (test LDH release − cell spontaneous release)/(cell maximal release − cell spontaneous release).

The hemolytic activity was measured as previously described [29]. One hundred microliters (100 μL) sterilized saline was added to each well of a 96-well cell plate. A 0.1-mL aliquot of the supernatant from a given strain was added to the first well, followed by a serial twofold dilution, with addition of 100 μL of 2% sheep red blood cells (RBCs). The 2% RBCs added with an equal volume of sterilized saline and water served as negative and positive controls, respectively. The plate was incubated at 37 °C for 1 h and placed at 4 °C overnight. The unlysed cells were precipitated by centrifugation. One hundred microliters of the supernatants were separated and transferred to a new 96-well plate, and the hemoglobin released was measured at OD_540_. The hemolytic activity was expressed as the reciprocal of the highest dilution of the culture filtrates that lead to the lysis of exceeding 50% RBCs.

The protease activity was measured as previously described [30]. Briefly, 250 μL of the supernatants were mixed with an equal volume of 0.5% (wt/vol) casein in 50 mM Tris–HCl (pH 8.0). After incubation at 37 °C for 2 h, the mixture was added to 500 μL pre-cooled 10% (wt/vol) trichloroacetic acid (TCA) and placed on ice for 30 min to precipitate the proteins. Then 500 μL of the supernatant was collected after centrifugation and mixed with an equal volume of 1 M NaOH. The absorbance was measured at OD_440_.

### Western blot for hemolysin co-regulated protein (Hcp) and NorV protein

Western blot was performed to determine the levels of Hcp protein expression and secretion in *A. hydrophila* wild-type, Δ*norV* and CΔ*norV* strains as described previously [31]. Briefly, both the cell pellets and culture supernatants from a given strain were collected and treated with 5× SDS-PAGE buffer. Aliquots of the samples were subjected to SDS-PAGE and electrophoretically transferred to NC membranes. The membranes were incubated with anti-Hcp, or anti-NorV, or anti-GroEL polyclonal antiserum followed by horseradish peroxidase (HRP)-conjugated goat anti-rabbit IgG. Antibody-antigen complexes were detected using an ECL Pico-detect kit (CMCTAG, USA) and ChemiDoc™ Touch imaging system (Bio-Rad, USA).

### qRT-PCR analysis for *hcp* and *norV* genes

The mRNA levels of *hcp* and *norV* genes in *A. hydrophila* were measured using qRT-PCR. Bacterial RNA was isolated with an E.Z.N.A. bacterial RNA isolation kit (Omega, Beijing, China). Then cDNA was synthesized in triplicate using HiScript qRT SuperMix (Vazyme Biotech). The cDNA amplification was manipulated using AceQ qPCR SYBR Green kit (Vazyme Biotech) in the Applied Biosystems StepOnePlus™ Real-Time PCR System (Thermo Fisher Scientific, USA). All procedures above were performed according to the manufacturer’s instructions. The internal housekeeping gene *recA* was used as the reference, and the acquired cycle threshold (CT) of each gene was normalized. The fold-change of mRNA levels was calculated using the 2^−ΔΔCT^ method [32]. The primers used are listed in Additional file 1.

### Bacterial resistance to predation by *T. thermophila*

The anti-predation ability of *A. hydrophila* was expressed as the relative survival of bacteria after co-cultured with *T. thermophila* [15]. Briefly, *A. hydrophila* (1 × 10^9^ CFU/mL) and *T. thermophila* SB210 (2 × 10^5^ cells/mL) in TBSS (2 mM KCl, 1 mM CaCl_2_, 0.5 mM MgCl_2_, and 1 mM Tris [pH 6.8–7.2]) were well mixed in equal volume. One hundred microliters of the mixture was transferred to each well of a 96-well plate. Meanwhile, *A. hydrophila* and SB210 mixed with same volume of TBSS respectively acted as the controls. TBSS alone served as the blank control. The plate was placed at 28 °C without shaking for 12 h, and the bacterial population was measured at OD_450_. The relative survival of bacteria was expressed as the OD_450_ value of the bacteria co-cultured with *T. thermophila* divided by that of bacteria grown alone at 12 h. The absorbance of *T. thermophila* cells was negligible [33]. The assay was duplicated in quadruplicate in three independent experiments.

### Determination of intracellular growth efficiency

The intracellular growth efficiency was determined using RAW264.7 macrophages as previously described [34]. *A. hydrophila* wild-type, Δ*norV* and CΔ*norV* strains grown to logarithmic phase were washed and resuspended in fresh serum-free DMEM. The RAW264.7 macrophages cultured in 24-well plate were infected with *A. hydrophila* at a multiplicity of infection (MOI) of 1:1 for 1 h at 37 °C. Extracellular bacteria were removed by washing and addition of DMEM containing gentamicin. To measure the intracellular bacterial survival rate, cells were sampled 1 h after the interaction with antibiotics (time point 0) and subsequently at a regular intervals of 20 min. Infected macrophages were washed three times with PBS and then treated with 0.1% (vol/vol) Triton X-100 for 10 min to fully lyse the cells and release intracellular bacteria. The CFU of intracellular bacteria was quantified using LB agar plates. The relative survival rate over time was calculated as follows: (average CFU at a specific time point/average CFU at time point 0) × 100.

### Determination of the bacterial median lethal dose (LD_50_)

The LD_50_ assay was performed using a zebrafish model as described by Pang et al. [33]. Zebrafish were provided by the Pearl River Fishery Research Institute, Chinese Academic of Fishery Science. *A. hydrophila* grown to logarithmic phase were washed three times and adjusted to 5 × 10^8^ CFU/mL, followed by a serial tenfold dilution with sterilized PBS. The zebrafish were divided into seven groups for each strain and each group consisted of ten zebrafish. The zebrafish were intraperitoneally injected with 20 μL of each dilution (10^1^ to 10^7^ CFU). Meanwhile, another ten zebrafish were injected with 20 μL of sterile PBS as the negative control. LD_50_ studies were carried out in triplicate for all of the strains. The numbers of dead fish showing clinical symptoms were recorded within 7 days. The LD_50_ values were calculated by the Reed and Muench method [35].

### Statistical analyses

The statistical analyses in this study were performed using the GraphPad Prism version 5 software. Student’s *t* test was used to analyze the difference between the Δ*norV* mutant and the wild type strain. Tukey’s multiple comparisons were preformed to analyze the *norV* mRNA levels of *A. hydrophila* NJ-35 grown under various conditions using one-way analysis of variance (ANOVA) with 95% confidence intervals. *P*-value < 0.05 was considered as a significant difference.

## Results

### NO activates the expression of *norV* gene

**Figure 1:**
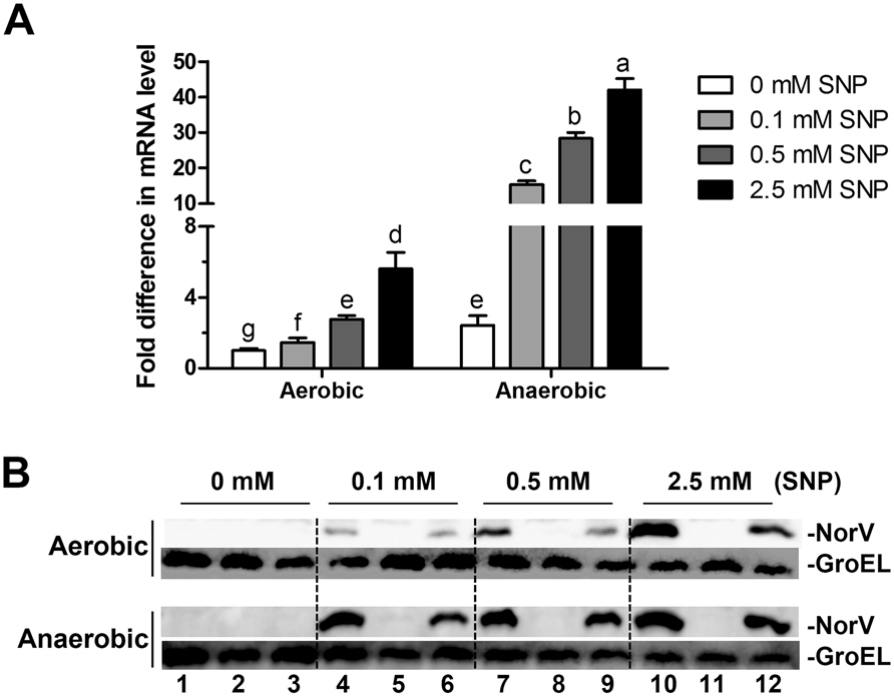
**The expression of** ***norV*** **in response to NO donor (sodium nitroprusside, SNP) under aerobic and anaerobic conditions.** The *A. hydrophila* strains were cultured to OD_600_ of 0.8 with various concentrations of NO donor under aerobic and anaerobic conditions. **A** The mRNA level of *norV* in the wild type strain determined by qRT-PCR. The expression value of *norV* without SNP treatment in the aerobic condition was normalized to 1.0. Values are means ± SD from three biological replicates. Different uppercase letters (a–g) indicate significant differences (*P* < 0.05) among different treatments. **B** The expression levels of NorV protein detected by Western blot. Lines 1, 4, 7, 10 represent the wild type strain NJ-35; Lines 2, 5, 8, 11 represent the Δ*norV* mutant; and Lines 3, 6, 9, 12 represent the complemented CΔ*norV* strain.

To determine how the *norV* gene in *A. hydrophila* responds to NO exposure, qRT-RCR analysis and Western blot were preformed. As shown in Figure 1, the transcription and expression levels of *norV* were increased when cells were exposed to SNP in a dose-dependent manner under both aerobic and anaerobic conditions, showing that *norV* could be activated by NO exposure regardless of the presence or absence of oxygen. Notably, the mRNA level of *norV* was significantly enhanced under anaerobic conditions compared to that under aerobic conditions (*P* < 0.001), suggesting that *norV* expression to some extent may be inhibited by oxygen.

### NorV affords protection against NO-mediated growth inhibition

**Figure 2:**
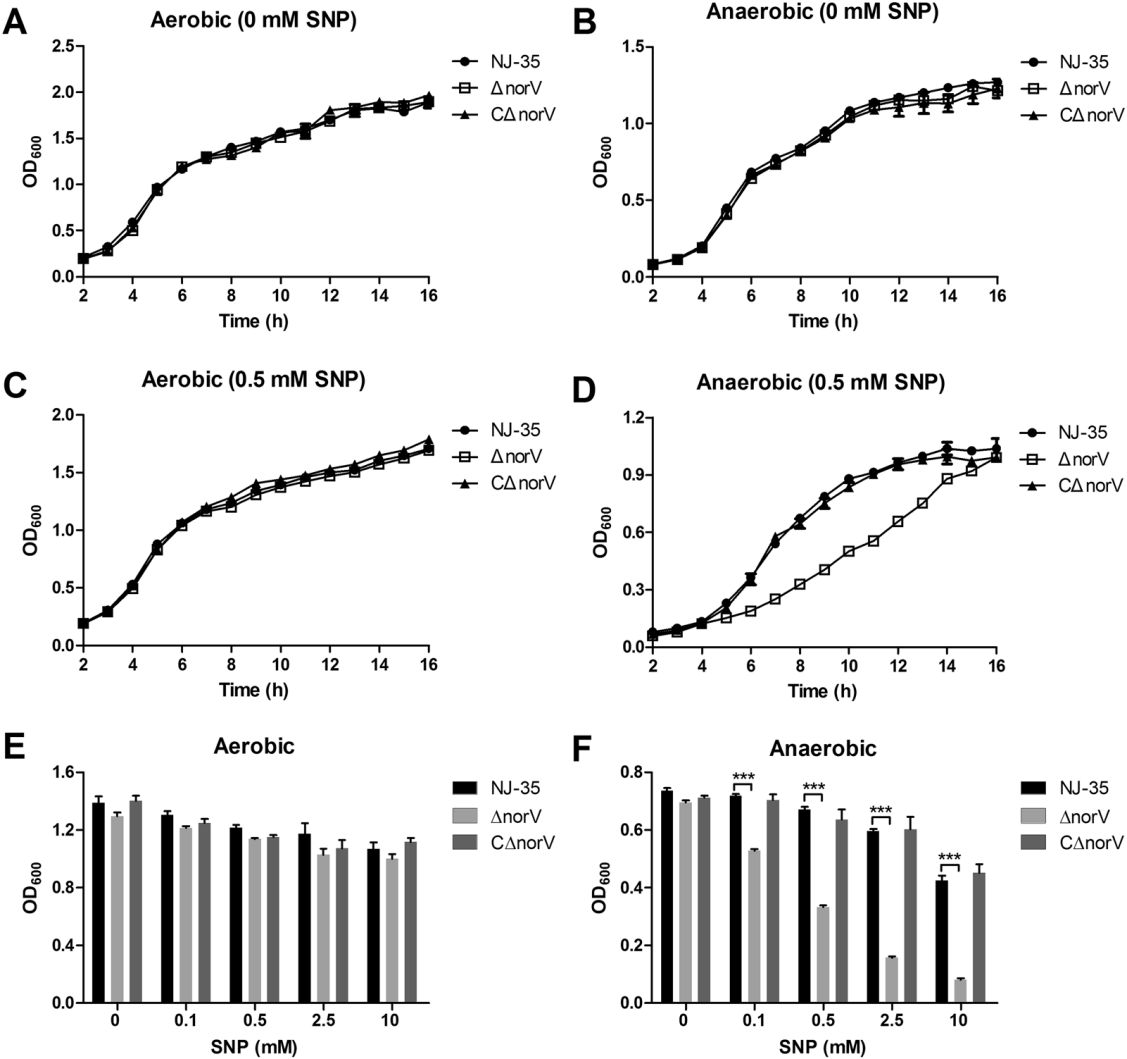
**Growth ability of the wild-type, Δ*norV*** **mutant, and complemented CΔ*norV*** **strains exposed to NO donor.** Growth curves of *A. hydrophila* strains were determined under aerobic (**A)** and anaerobic (**B)** conditions without SNP treatment. After the addition of 0.5 mM SNP, the growth yields of *A. hydrophila* strains under aerobic (**C)** or anaerobic (**D)** conditions were detected by measuring the OD_600_ values. NO growth inhibition assay was performed in bacteria grown for 8 h at 28 °C in LB broth containing various concentrations of SNP under aerobic (**E)** or anaerobic (**F)** conditions. Values are the means ± SD from three biological replicates. ***P < 0.001.

To examine how NorV in *A. hydrophila* responds to NO, we determined the growth difference between the wild-type and Δ*norV* mutant strains exposed to NO donor. As shown in Figures 2A and B, the growth of the wild-type strain and Δ*norV* mutant showed no difference both under the aerobic and anaerobic conditions in the absence of NO. In the aerobic cultures containing SNP, the growth rates of Δ*norV* were comparable to the wild type strain (Figures 2C and E). However, the Δ*norV* mutant displayed a reduction of anaerobic growth in response to SNP in a dose-dependent manner (Figures 2D and F). The growth capacity was restored in the complemented strain CΔ*norV*.

### The *norV* is involved in cytotoxicity, hemolytic and protease activities

**Figure 3:**
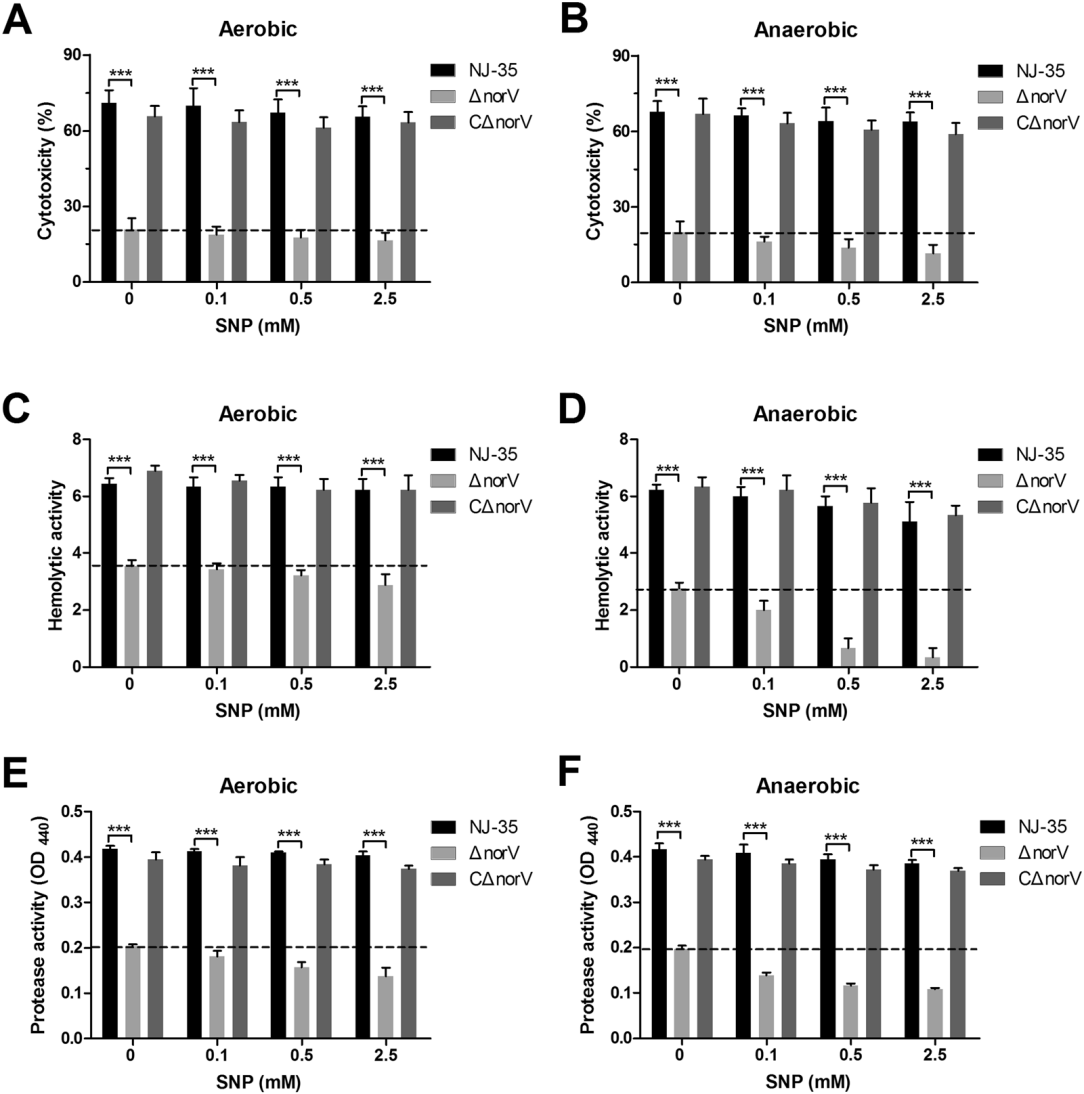
**Cytotoxicity, hemolytic and protease activities of the wild-type, Δ*norV*** **mutant, and complemented CΔ*norV*** **strains grown under various conditions.** Cytotoxicity was expressed as LDH release of RAW264.7 macrophages induced by culture supernatants collected from bacteria grown under aerobic (**A)** and anaerobic (**B)** conditions after exposure to various concentrations of SNP. The hemolytic activities of each strain grown under aerobic (**C**) and anaerobic (**D**) conditions after treating with various concentrations of SNP were expressed as the dilution-fold of the *A. hydrophila* culture filtrates that led to the lysis of 50% erythrocytes. The protease activities of *A. hydrophila* strains cultured in LB media containing various concentrations of SNP under aerobic (**E**) and anaerobic (**F**) conditions were measured at OD_440_ with azocasein as the substrate. Values are the means ± SD from three biological replicates. ***P < 0.001.

To determine whether NorV is associated with the production of extracellular products, the cytotoxicity, hemolytic and protease activities were determined. As shown in Figures 3A and B, the culture filtrates collected from the Δ*norV*, grown both under aerobic and anaerobic conditions, produced significantly lower injury to macrophages than those of the wild-type strain (*P* < 0.001). In addition, the Δ*norV* mutant displayed poorer hemolytic activity than the wild-type strain when cultured both under aerobic and anaerobic conditions (*P* < 0.001) (Figures 3C and D). Similarly, the protease activity of Δ*norV* was also markedly decreased (*P* < 0.001) (Figures 3E and F). Notably, the cytotoxicity, hemolytic and protease activities of Δ*norV* mutant when exposed to NO under anaerobic conditions were decreased in a dose-dependent manner, whereas their activities in the wild-type strain showed no obvious difference. Moreover, the hemolytic activity of the Δ*norV* mutant under anaerobic conditions was lower than that under aerobic conditions. High concentrations of NO could exert reduced effect on hemolytic activity of the wild-type. All the activities of extracellular products were restored in the CΔ*norV*.

### The *norV* is diversely involved in production and secretion of Hcp protein

**Figure 4:**
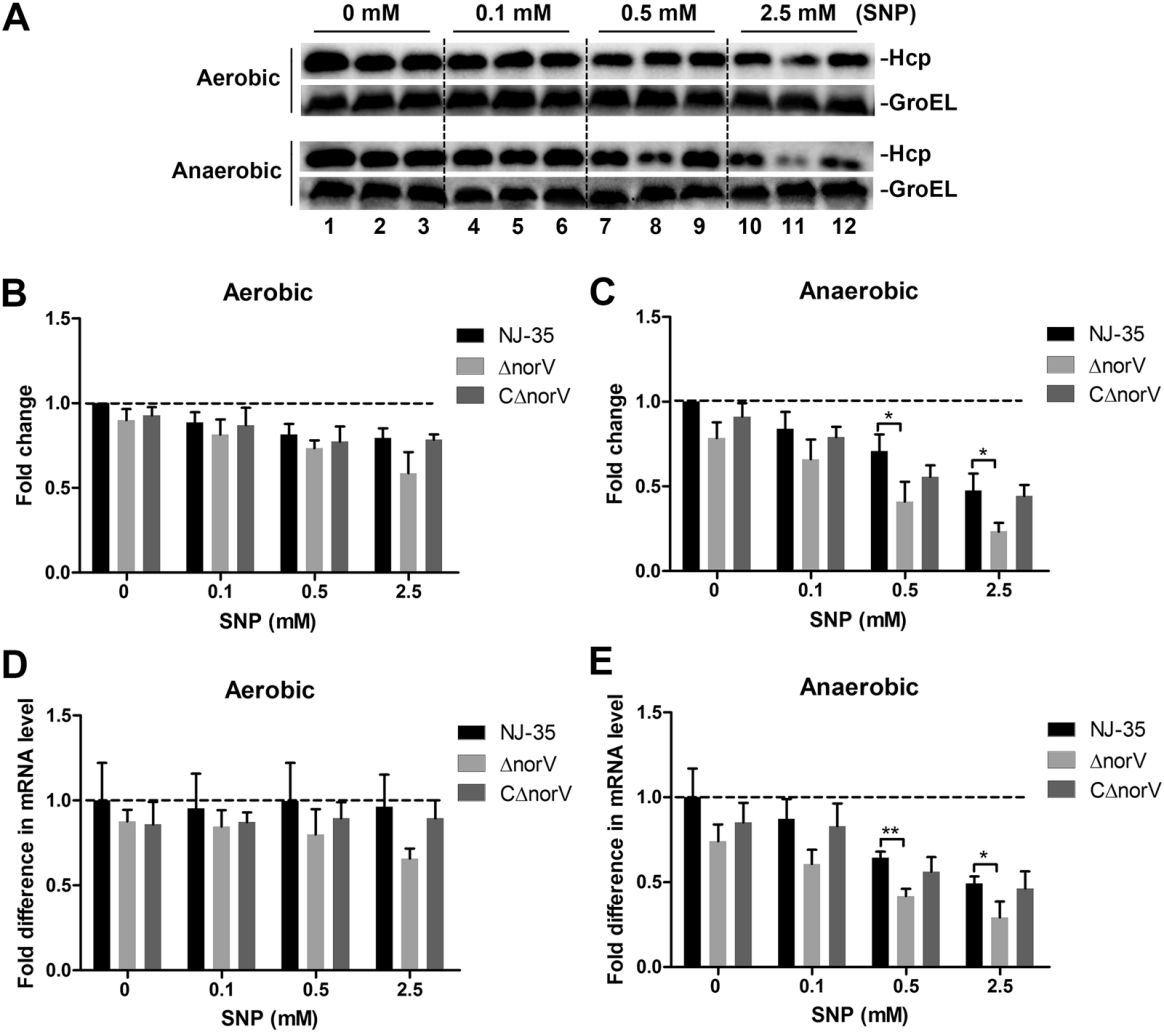
**Effects of** ***norV*** **on Hcp protein expression. A** The Hcp proteins in the whole cells of bacteria grown under aerobic and anaerobic conditions exposing to various concentrations of SNP were determined by Western blot. Polyclonal anti-Hcp antibodies were used to measure the production of Hcp and anti-GroEL antibodies served as internal reference. Lines 1, 4, 7, 10 represent the wild type strain NJ-35; Lines 2, 5, 8, 11 represent the Δ*norV* mutant; and Lines 3, 6, 9, 12 represent the complemented CΔ*norV* strain. The relative changes of Hcp production of bacteria cultured under aerobic (**B**) and anaerobic (**C**) conditions are given between the blots. The intensities of the Hcp and GroEL bands were measured and Hcp was equalized to that of GroEL. The relative mRNA levels of *hcp* gene in *A. hydrophila* strains grown under aerobic (**D**) and anaerobic (**E**) conditions after treated with various concentrations of SNP were determined by qRT-PCR. Values are the means ± SD from three biological replicates. *P < 0.05, **P < 0.01.

**Figure 5:**
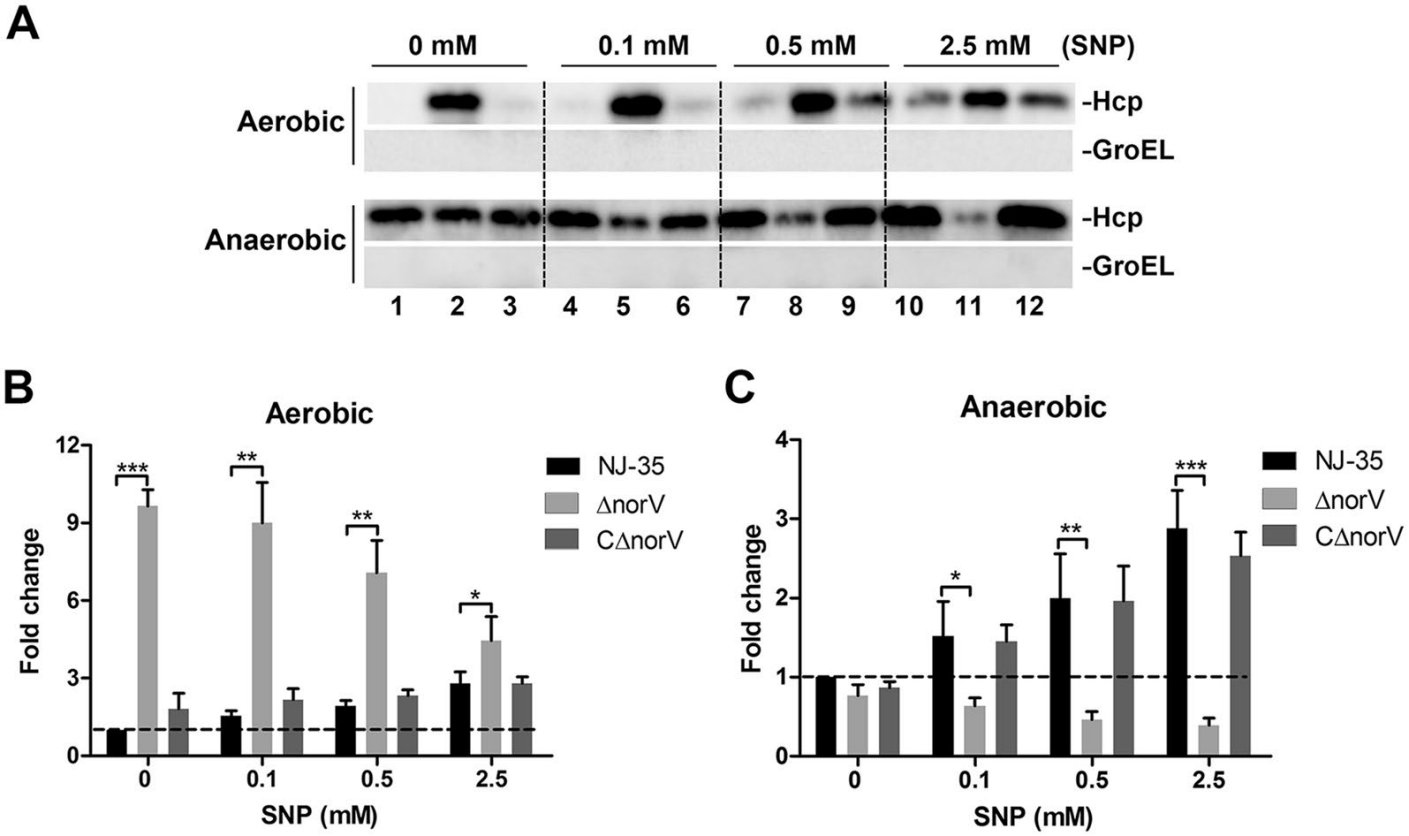
**The** ***norV*** **is diversely involved in secretion of Hcp protein. A** Hcp proteins in the culture supernatants of bacteria grown under aerobic and anaerobic conditions after exposure with various concentrations of SNP were determined by Western blot. Polyclonal anti-Hcp antibodies were used to measure the secretion of Hcp and anti-GroEL antibodies served as internal reference. Lines 1, 4, 7, 10 represent the wild type strain NJ-35; Lines 2, 5, 8, 11 represent the Δ*norV* mutant; and Lines 3, 6, 9, 12 represent the complemented CΔ*norV* strain. The relative changes of Hcp secretion of bacteria grown under aerobic (**B**) and anaerobic (**C**) conditions are given between the blots. Values are the means ± SD from three biological replicates. *P < 0.05, **P < 0.01, ***P < 0.001.

To determine whether the *norV* gene is involved in regulating the function of T6SS in response to NO, the expression and secretion of Hcp in *A. hydrophila* wild-type, Δ*norV* mutant and CΔ*norV* strains were examined. When exposed to NO, Hcp expression showed no obvious difference between Δ*norV* mutant and wild-type strain under aerobic conditions (Figures 4A and B). However, under anaerobic conditions, Hcp expression displayed a decreased tendency after exposure to NO and that of Δ*norV* mutant was significantly lower than the wild-type strain (*P* < 0.05) with SNP at concentrations of 0.5 mM and 2.5 mM (Figures 4A and C). The mRNA levels of *hcp* were consistent with protein expression results (Figures 4D and E). However, as shown in Figure 5A, NO exposure led to an increase in Hcp secretion of the wild-type strain but a decrease in Δ*norV* mutant both under aerobic and anaerobic conditions. Hcp secretion of Δ*norV* mutant was higher in the aerobic environment but poorer under anaerobic conditions than the wild-type strain (Figures 5B and C). The cytoplasmic chaperone protein GroEL was not detectable in the supernatants, demonstrating that the Hcp detected was the result of bacterial secretion but not cell lysis.

### The *norV* contributes to resisting predation by *T. thermophila*

In order to study whether *norV* inactivation exerts influence on the interaction between *A. hydrophila* and *T. thermophila*, the relative survivals of the wild type strain NJ-35 and its *norV* derivatives were detected after co-culture with *T. thermophila* for 12 h. The relative survival of Δ*norV* mutant (51.83 ± 4.02%) exhibited a significant reduction (*P* = 0.0054) compared to that of the wild-type strain (68.10 ± 3.20%). The anti-protistan predation level was restored in the complemented strain (63.72 ± 4.73%).

### The *norV* enhances the intracellular survival of *A. hydrophila* in macrophages

**Figure 6:**
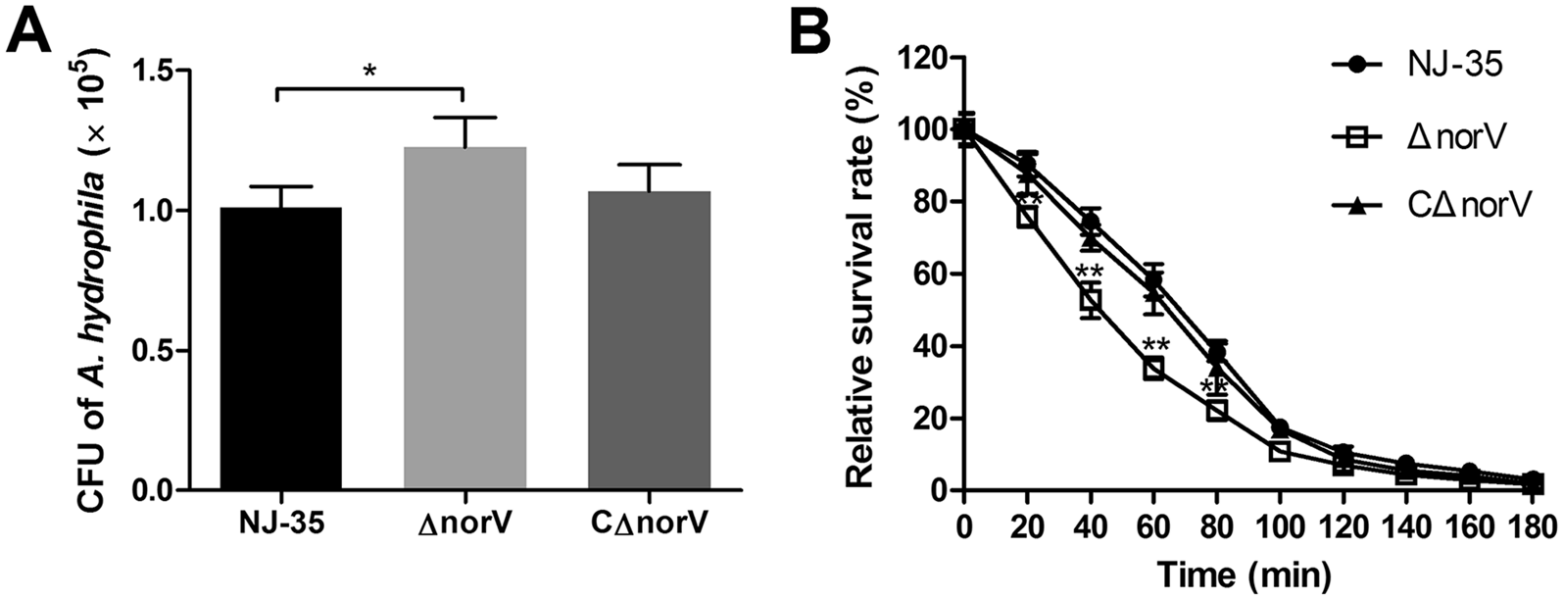
**Survival efficiency of the wild-type, Δ*norV*** **mutant, and complemented CΔ*norV*** **strains in RAW264.7 macrophages. A** CFU of intracellular bacteria at 1 h post-infection (time point 0). **B** Intracellular survival rate of *A. hydrophila* in macrophages. Phagocytosis was allowed to proceed for 1 h at a MOI of 1.0. The CFU of intracellular bacteria was estimated every 20 min. The relative survival was calculated by dividing the mean CFU at a specific time point by the CFU at time point 0. The survival rate at time point 0 was arbitrarily set to 100%. Data are presented as the mean ± SD of three independent experiments. *P < 0.05, **P < 0.01.

The bacteria within RAW264.7 macrophages were quantified by CFU counts. Compared with the wild-type, more intracellular viable bacteria of the Δ*norV* mutant were observed, suggesting that the Δ*norV* is more susceptible to phagocytosis by macrophages (Figure 6A). Moreover, the Δ*norV* mutant displayed impaired survival rate in macrophages in a time-dependent manner (Figure 6B), indicating that the Δ*norV* engulfed was more likely to be cleared by macrophages.

### The *norV* is required for the virulence of *A. hydrophila* in zebrafish

To investigate the effect of *norV* on the virulence of *A. hydrophila*, zebrafish were injected intraperitoneally with the wild-type or Δ*norV* mutant strain. The mortality of zebrafish was recorded within 7 days after infection. The LD_50_ value of the Δ*norV* mutant (10^4.09^ ± 10^3.03^ CFU) was 87-fold higher than that of the wild-type strain (10^2.16^ ± 10^1.37^ CFU), indicating a significant reduction in the virulence of the Δ*norV* mutant (*P* < 0.0001). Virulence was almost restored when the CΔ*norV* (10^2.75^ ± 10^1.89^ CFU) was used to infect zebrafish.

## Discussion

Nitric oxide is an important component of host defence against invading pathogenic bacteria [19, 25, 27, 36, 37]. It is likely to be encountered by bacteria in diverse environments. To overcome the deleterious effects of NO, bacteria have evolved multiple mechanisms to protect against NO stress, for example, detoxifying NO via the NorV [38]. In this study, we used SNP as NO donor and investigated the biological functions of NorV in *A. hydrophila*. Before entering the study, we evaluated the kinetics of NO release by SNP using Griess assay and found NO could be released continuously for more than 10 h (Additional file 2). Based on the releasing dynamic, SNP of 2.5 mM could produce a concentration of NO_2_^−^ of about 10 μM in LB medium. Shimizu et al. [27] revealed that the NO_2_^−^ concentration in the supernatants of infected RAW264.7 cells by *E. coli* could be up to nearly 20 μM after 10 h post-infection. Thus, it is possible that bacteria will have an opportunity to encounter relatively high amount of NO during infection. The SNP concentrations used in this study are, therefore, biologically relevant.

NorV has been known for the detoxification of NO under anaerobic, but not aerobic conditions [19, 20]. However, there is evidence that the *norV* promoter can also be activated by NO exposure during aerobic growth [39]. To determine whether the activation of NorV correlated with the presence or absence of oxygen, we examined its mRNA and protein expression under aerobic and anaerobic conditions. Our data demonstrated that the transcription of *norV* in *A. hydrophila* was regulated in a NO-dependent manner in both conditions, and NorV expression would be activated by NO exposure with or without oxygen. Surprisingly, however, NorV could only exert a protective growth effect by eliminating NO under anoxic conditions. Hence, these findings prompt us to explore whether and which physiological role NorV plays in the presence of oxygen in *A. hydrophila*.

Both environmental oxygen and NO signals are able to reprogram gene expression and to stimulate bacterial secretion systems, effector molecules, or toxins to promote survival and resist injury [36, 37, 40]. Extracellular proteins such as hemolysins, proteases and cytotoxins are belived to exhibit potential pathogenicity in *Aeromonas* spp. [41, 42]. In this study, we demonstrated for the first time that the *norV* gene plays important roles in the secretion of bacterial extracellular products. The activities of ECPs from the Δ*norV* strain remained aerobically unaffected by NO but anaerobically decreased in a NO dose-dependent manner. This phenomenon leads us to speculate that NO can anaerobically inhibit the production of ECPs and NorV serves as a NO scavenger to minimize the inhibition effect of NO. Notably, however, the activities of ECPs in Δ*norV* mutant were significantly lower than those in the wild-type strain even without exposure to NO, indicating that NorV could regulate the production of ECPs beyond depending on its nitric oxide reductase activity. To exclude the potential polar effect of *norV* disruption on the production of ECPs, we studied the transcriptional levels of both upstream (*norR*) and downstream (*norW*) genes of the *norV* deletion region. The data showed that the inactivation of *norV* did not affect the transcription of either *norR* or *norW* (Additional file 3), indicating no polar mutation had occurred. In *A. hydrophila*, type II secretion system (T2SS) has been known to be essential for the translocation of ECPs from the periplasm into the extracellular milieu [43, 44]. Thus, it is speculated that NorV might affect the secretion of extracellular proteins through T2SS. In this regard, it may be of interest to further investigate the relationship of *norV* and T2SS.

In some Gram-negative bacteria, T6SS is often involved in virulence-related mechanisms and environmental competitive fitness [45, 46]. Intriguingly, similar to the impact on ECPs, we found that NorV anaerobically contributed to Hcp production in a NO-dependent fashion. Compared to the wild-type, Hcp secretion in the Δ*norV* mutant was higher under oxic conditions but lower under anoxic conditions, indicating that NorV is involved in Hcp secretion and the involvement effect might be strongly related to the presence of oxygen. Oxygen is a constantly changing environmental parameter in bacterial infections [47]. To adapt to the variable oxygen conditions, bacteria have to convert metabolic strategies [40]. A study from Babujee et al. [40] has demonstrated that oxygen limitation was associated to varying degrees with the Hcp production in *Dickeya dadantii* and *Pectobacterium atrosepticum*. Also, it should be noted that Hcp secretion in the wild type strain was increased in a NO dose-dependent manner but decreased in the Δ*norV* mutant. This indicates that the promotion on Hcp secretion by NO may positively correlate with NorV, and the NO-dependent decrease of Hcp secretion in the Δ*norV* mutant probably imply an indirect effect. Some bacteria have been reported to develop other NO defense mechanisms, including flavohemoglobin (Hmp) [48] and cytochrome *c* nitrite reductase (NrfA) [49] in addition to the NorV. However, we demonstrated that the inactivation of *norV* gene did not affect the transcription levels of *hmp* or *nrfA* (Additional file 4). We can, however, not exclude the possibility that the inactivation of *norV* may affect other unknown NO defense systems. Also, various classes of regulators sensitive to environmental cues have been demonstrated to specifically modulate the T6SS activity, for example, iron, σ^54^-dependent activator proteins and surface association [50]. We could not determine whether these regulators may be involved in the Hcp secretion in *A. hydrophila*. Maybe future transcriptomic study will be helpful to explain the molecular basis of the altered Hcp secretion due to NorV.

In the present study, we also determined that *norV* gene had ability to confer a protective advantage for *A. hydrophila* in resisting predation by *Tetrahymena* and improving the bacterial survival within macrophages, and made an important contribution to bacterial virulence in zebrafish. NorV was confirmed to play an important role for the survival of EHEC within macrophages by eliminating NO produced by iNOS [27]. However, we believe the mechanism by which NorV increased *A. hydrophila* survival in macrophages cannot be due to its NO consumption activity. Some previous studies have indicated that NO could only be produced by macrophages after 8 h post-infection [27, 51], but in our study, *A. hydrophila* was almost cleared by cells within 3 h. Additional regulatory mechanisms may account for the contribution of *norV* in bacterial survival within macrophages.

In summary, our results reveal that *norV* gene has various effects on the virulence-associated traits of *A. hydrophila.* Regulation of these virulence traits in response to external conditions reflects that this bacterium has the ability to regulate its cellular activity in order to adapt to the environment in which it is growing.

## Supplementary information

**Additional file 1. Primers used in this study.**

**Additional file 2. Kinetics of NO release in LB medium by SNP.** (A) Standard curve between NO_2_^−^ concentration and optical density (OD_540_) was determined by Greiss assay using sodium nitrite (NaNO_2_) as a standard. (B) LB media containing various concentrations of SNP were incubated at 28 °C. The concentration of NO_2_^−^ in LB medium was evaluated by Greiss assay over time. Data are presented as the mean ± SD of three independent experiments.

**Additional file 3. The mRNA levels of upstream (*norR*) and downstream (*norW*) genes of the** ***norV*-deletion region.** *A. hydrophila* wild-type, Δ*norV* mutant, and complemented CΔ*norV* strains were cultured with or without SNP to OD_600_ of 0.8 both under aerobic and anaerobic environment. Cells were collected and the RNA was extracted. The expression values of *norR* or *norW* gene were determined by qRT-PCR. The relative change in corresponding gene expression was normalized to the expression of a housekeeping gene (*recA*) and calculated by the 2^−ΔΔCT^ method. The fold changes in mRNA level of genes in Δ*norV* mutant and complemented CΔ*norV* strain were normalized to that in *A. hydrophila* NJ-35 under the same condition. (A) The mRNA levels of *norR* under aerobic conditions. (B) The mRNA levels of *norR* under anaerobic conditions. (C) The mRNA levels of *norW* under aerobic conditions. (D) The mRNA levels of *norW* under anaerobic conditions. Values are the means ± *SD* from three biological replicates.

**Additional file 4. The mRNA levels of flavohemoglobin coding gene** ***hmp*** **and cytochrome** ***c*** **nitrite reductase coding gene** ***nrfA***. *A. hydrophila* wild-type, Δ*norV* mutant, and complemented CΔ*norV* strains were cultured with or without SNP to OD_600_ of 0.8 both under aerobic and anaerobic environment. Cells were collected and the RNA was extracted. The expression values of *hmp* and *nrfA* genes were determined by qRT-PCR. The relative change in corresponding gene expression was normalized to the expression of a housekeeping gene (*recA*) and calculated by the 2^−ΔΔCT^ method. The fold changes in mRNA level of genes in Δ*norV* mutant and complemented CΔ*norV* strain were normalized to that in *A. hydrophila* NJ-35 under the same condition. (A) The mRNA levels of *hmp* under aerobic conditions. (B) The mRNA levels of *hmp* under anaerobic conditions. (C) The mRNA levels of *nrfA* under aerobic conditions. (D) The mRNA levels of *nrfA* under anaerobic conditions. Values are the means ± *SD* from three biological replicates.

## Data Availability

All data generated or analysed during this study are included in this published article and its Additional files.

## Abbreviations

NorV: nitric oxide reductase
NO: nitric oxide
iNOS: inducible nitric oxide synthase
SNP: sodium nitroprusside
OD: optical density
LB: Luria–Bertani
Amp^r^: ampicillin resistance
Cm^r^: chloramphenicol resistance
PCR: polymerase chain reaction
CFU: colony forming unit
DMEM: Dulbecco’s Modified Eagle’s Medium
FBS: fetal bovine serum
LDH: lactate dehydrogenase
MOI: multiplicity of infection
PBS: phosphate buffered saline
ECPs: extracellular products
RBCs: red blood cells
Hcp: hemolysin co-regulated protein
TCA: trichloroacetic acid
GroEL: heat shock protein Hsp60
LD_50_: median lethal dose
T2SS: type II secretion system
T6SS: type VI secretion system
