# Supplementary material for: Diverse effects of nitric oxide reductase NorV on Aeromonas hydrophila virulence-associated traits under aerobic and anaerobic conditions
Source: Vet Res. 2019 Sep 23;50:67. doi: 10.1186/s13567-019-0683-6 (PMC6755692; doi:10.1186/s13567-019-0683-6)
Supplement: Supplementary file 1 — Additional file 1. Primers used in this study. [file 13567_2019_683_MOESM1_ESM.doc]

**Additional file 1 Primers used in this study**

| Primer | Sequence(5’-3’) | Function | |
| --- | --- | --- | --- |
| *norV* -P_1_ | CAGGTCGACTCTAGAGGATCCCGAGACCTGTCCCGCACT (*Bam*H I) ^a^ | Construction of *norV* deletion mutant | |
| *norV* –P_2_ | AGGAACTGCCTGTGTATGAGCACAACAACGCA |
| *norV* –P_3_ | TGTGCTCATACACAGGCAGTTCCTCGAATGC |
| *norV* –P_4_ | GAGCTCGGTACCCGGGGATCCGCTCCTCGTCGCTGATGG (*Bam*H I) |
| *norV*-C1 | CCTACTCCGGCAGCGGT ^b^ | Construction of *norV* complemented strain combined with *norV* -P_1_ and *norV* –P_4_ | |
| *norV*-C2 | ACCGCTGCCGGAGTAGG ^b^ |
| *norV*-F | CAAGGAAGTGCTCGGTTT | Used for qRT-PCR | |
| *norV*-R | GTAGAAGAGGGTGATGCGA |
| *hcp* -F | CACAAGCCGTTCAAGTTCAC | Used for qRT-PCR | |
| *hcp* -F | GTCACTTTCGGCAGCATTTC |
| *recA*-F | CGACCCCATCTATGCCGC | Used for qRT-PCR | |
| *recA*-R | CCATCTCACCTTCGATTTCCG |  | |
| *norR*-F | CGCAGTTGAGGTAAACCA | Used for qRT-PCR |  |
| *norR*-R | CTGATCCTGGGAGAGACC |  |
| *norW*-F | TTCCTGCAACCCATAGTG | Used for qRT-PCR |  |
| *norW*-R | CGTCTTCACCTTCACCAG |  |
| *hmp*-F | CTACCGCATCAGCGTCAAAC | Used for qRT-PCR |  |
| *hmp*-R | CACCTCAATCTTGTCACCCG |  |
| *nrfA*-F | GATGAGAAAGGTGAGGATGGCT | Used for qRT-PCR |  |
| *nrfA*-R | GCATAGGGACGGGAGAGGT |  |

^a^ Restriction sites are underlined.

^b^ Point mutation sites are underlined.
