# Supplementary figures and images for: Diverse effects of nitric oxide reductase NorV on Aeromonas hydrophila virulence-associated traits under aerobic and anaerobic conditions
Source: Vet Res. 2019 Sep 23;50:67. doi: 10.1186/s13567-019-0683-6 (PMC6755692; doi:10.1186/s13567-019-0683-6)

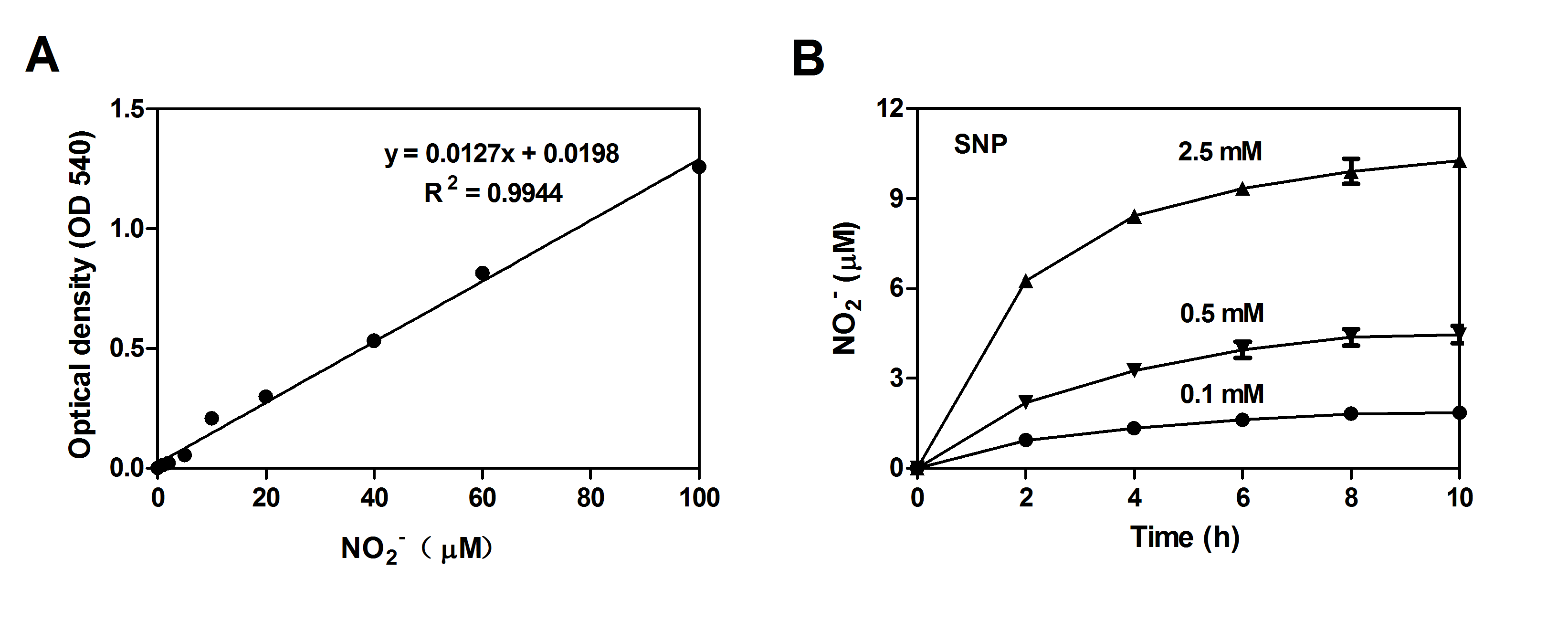

Supplement: Supplementary file 2 — Additional file 2. Kinetics of NO release in LB medium by SNP. (A) Standard curve between NO2− concentration and optical density (OD540) was determined by Greiss assay using sodium nitrite (NaNO2) as a standard. (B) LB media containing various concentrations of SNP were incubated at 28 °C. The concentration of NO2− in LB medium was evaluated by Greiss assay over time. Data are presented as the mean ± SD of three independent experiments. [file 13567_2019_683_MOESM2_ESM.tif]

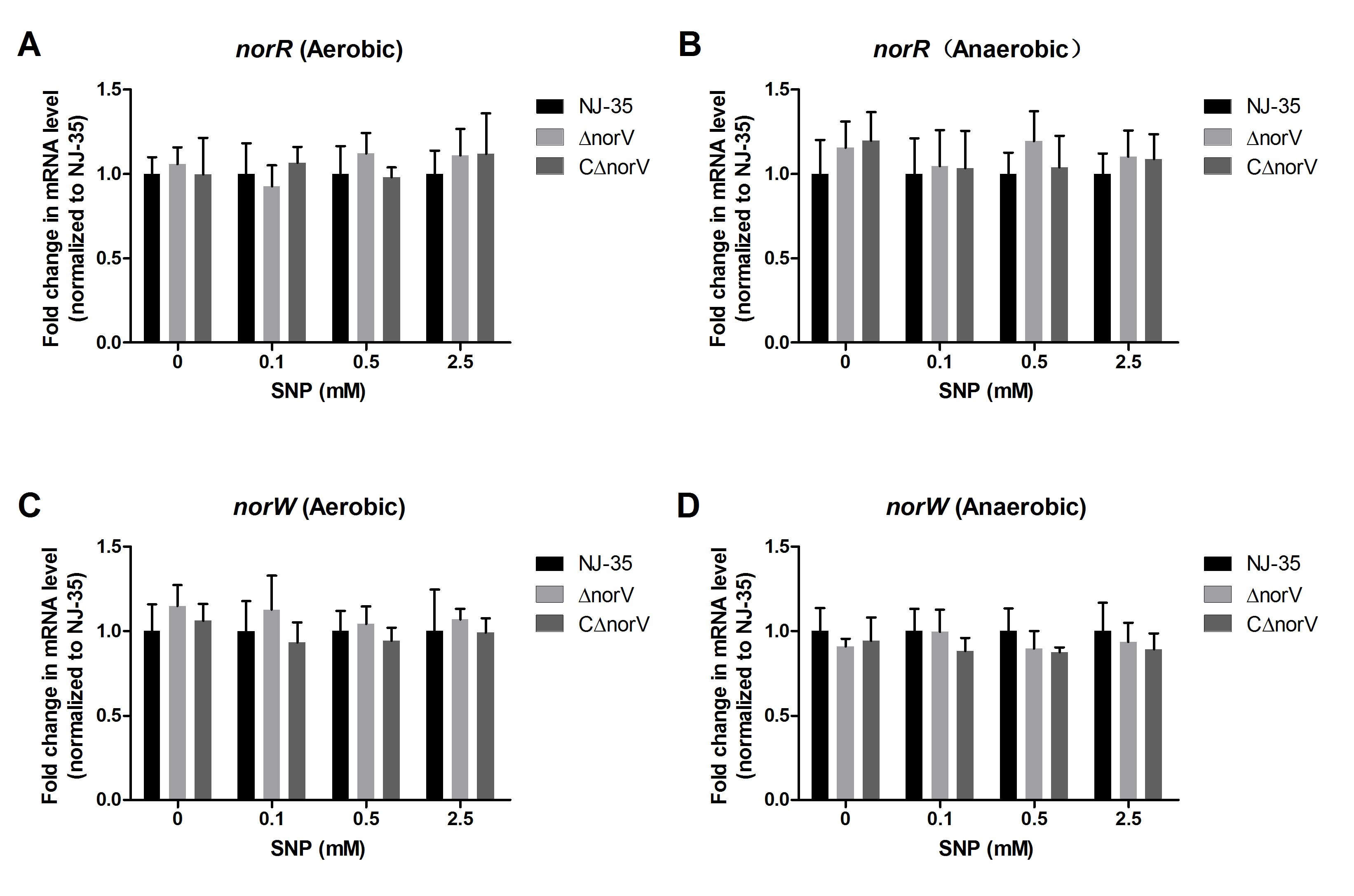

Supplement: Supplementary file 3 — Additional file 3. The mRNA levels of upstream (norR) and downstream (norW) genes of the norV-deletion region. A. hydrophila wild-type, ΔnorV mutant, and complemented CΔnorV strains were cultured with or without SNP to OD600 of 0.8 both under aerobic and anaerobic environment. Cells were collected and the RNA was extracted. The expression values of norR or norW gene were determined by qRT-PCR. The relative change in corresponding gene expression was normalized to the expression of a housekeeping gene (recA) and calculated by the 2−ΔΔCT method. The fold changes in mRNA level of genes in ΔnorV mutant and complemented CΔnorV strain were normalized to that in A. hydrophila NJ-35 under the same condition. (A) The mRNA levels of norR under aerobic conditions. (B) The mRNA levels of norR under anaerobic conditions. (C) The mRNA levels of norW under aerobic conditions. (D) The mRNA levels of norW under anaerobic conditions. Values are the means ± SD from three biological replicates. [file 13567_2019_683_MOESM3_ESM.tif]

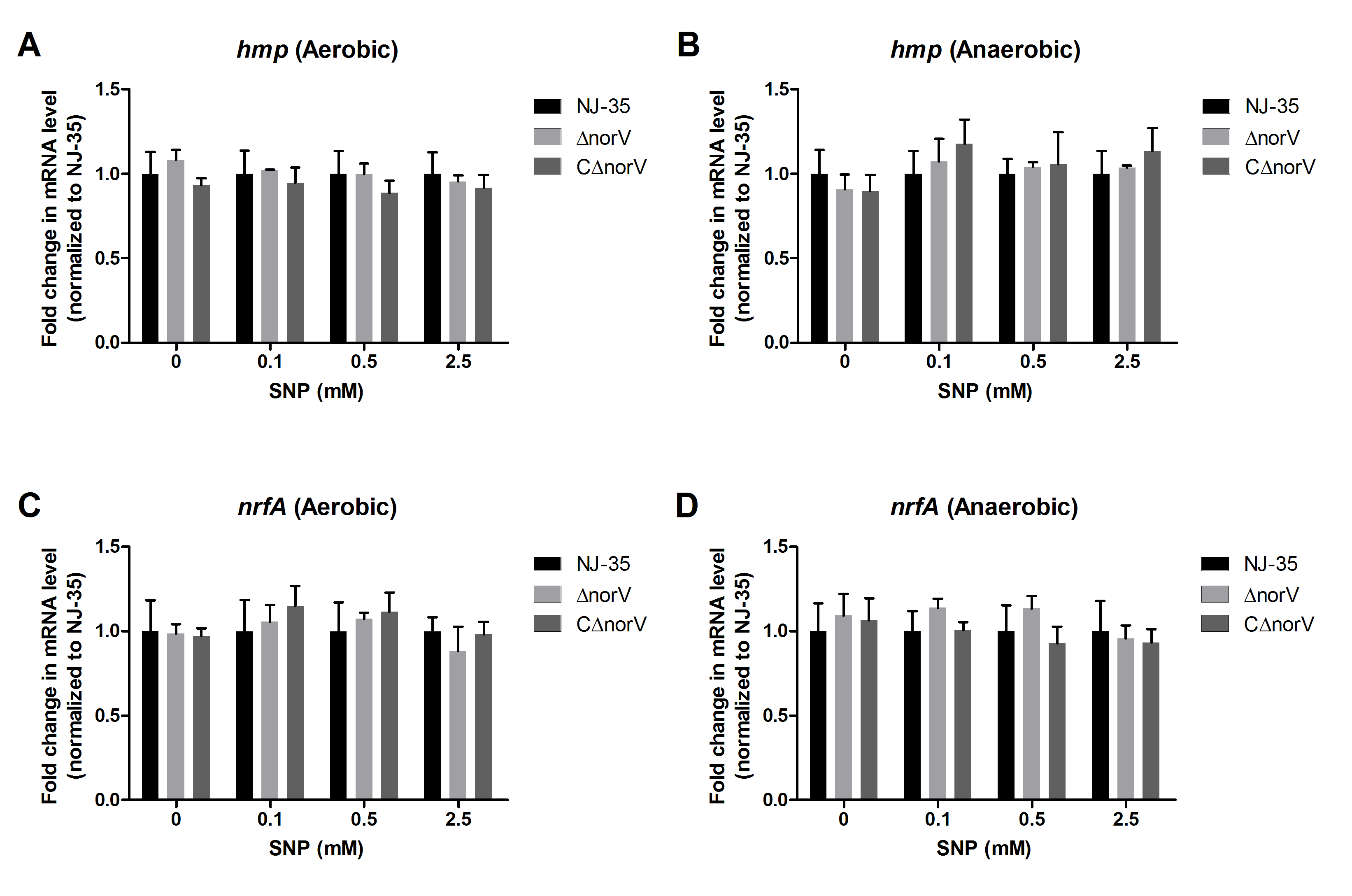

Supplement: Supplementary file 4 — Additional file 4. The mRNA levels of flavohemoglobin coding gene hmp and cytochrome c nitrite reductase coding gene nrfA. A. hydrophila wild-type, ΔnorV mutant, and complemented CΔnorV strains were cultured with or without SNP to OD600 of 0.8 both under aerobic and anaerobic environment. Cells were collected and the RNA was extracted. The expression values of hmp and nrfA genes were determined by qRT-PCR. The relative change in corresponding gene expression was normalized to the expression of a housekeeping gene (recA) and calculated by the 2−ΔΔCT method. The fold changes in mRNA level of genes in ΔnorV mutant and complemented CΔnorV strain were normalized to that in A. hydrophila NJ-35 under the same condition. (A) The mRNA levels of hmp under aerobic conditions. (B) The mRNA levels of hmp under anaerobic conditions. (C) The mRNA levels of nrfA under aerobic conditions. (D) The mRNA levels of nrfA under anaerobic conditions. Values are the means ± SD from three biological replicates. [file 13567_2019_683_MOESM4_ESM.tif]
